# Comparative evaluation of score criteria for dynamic Bayesian Network structure learning

**DOI:** 10.1371/journal.pone.0336250

**Published:** 2025-11-05

**Authors:** Aslı Yaman, Mehmet Ali Cengiz

**Affiliations:** 1 Department of Management Information Systems, Faculty of Economics and Administrative Sciences, Istanbul Arel University, Istanbul, Turkey; 2 Department of Mathematics and Statistics, College of Science, Imam Mohammad Ibn Saud Islamic University (IMSIU), Riyadh, Saudi Arabia; Federation University Australia, AUSTRALIA

## Abstract

Dynamic Bayesian Networks (DBNs) are probabilistic models with a directional structure employed to model temporal processes. Three approaches to DBN structure learning are constraint-based, score-based, and hybrid. The score criterion determined in the score-based and hybrid approach has a certain effect on structure learning and this study aims to examine their performance by diversifying the score criteria used in DBN structure learning in addition to the scores commonly used in the literature. Thus, the Akaike-based information criteria as Akaike Information Criterion (AIC), Consistent AIC (CAIC), Kullback-Leibler Information Criterion (KIC), AIC4, and the Bayesian-based information criteria as Bayesian Information Criterion (BIC), Adjusted BIC (BICadj), Haughton BIC (HBIC), BICQ were adapted into the DBN structure learning. The obtained results were discussed.

## Introduction

Bayesian Networks (BNs) use a directed acyclic graph to represent a joint probability distribution. BNs are widely preferred for combining different sources of knowledge (expert and/or data) and giving successful results in small and incomplete datasets [1].

They can be used for a wide range of tasks including prediction [2], anomaly detection [3], diagnostics [4], automated insight [5], reasoning [6], time series prediction [7], and decision-making under uncertainty [8,9]. More detailed information about BNs can be obtained from [10–13].

Despite their success in various domains, BNs are inadequate for analyzing temporal processes or datasets with cyclical relationships. Therefore, DBNs, as an advancement of BNs incorporating the concept of time, are used in modeling temporal processes. The DBN can be thought of as a network of BNs at multiple different time points, while the BNs represent the problem at a particular time point. With the DBN, both the causal relationships between the variables at a certain time point and the relationships between the variables at different time points can be analysed.

DBN’s common usage areas are feature recognition/detection [14], fault detection and diagnosis [15], risk analysis [16], analysing of gene networks [17], and forecasting in time series analysis [18].

The analysis of DBNs comprises two components. First, the network structure is generated in which variables and causality structures between variables are visualized, then the analysis is performed by obtaining the probability and parameter values over the known network structure. Therefore, it is thought that the more appropriate the network structure is generated for the dataset, the stronger the analysis will give. For this reason, this study mainly focused on determining of the network structure in DBNs.

Two approaches are available for determining the network structure in DBNs. First, the appropriate network structure for the dataset can be created using expert knowledge. Experts in the field can create the network structure by determining the cause-and-effect structures between the variables. However, this method may not always give reliable results. Because it may not always be possible to reach experts in the field, it may be difficult to determine the arcs in large datasets and/or may take a lot of time.

On the other hand, network structures can be obtained using the existing dataset. Determining the network structure through the application of algorithms to a dataset is known as structure learning. In DBNs, structure learning can be approached through three distinct methodologies. These methodologies include score-based, constraint-based, and hybrid algorithms. In constraint-based algorithms, the structures of relationships between variables are inferred through conditional independence tests. In score-based algorithms, a search method and a score function are determined and the network structure is determined so that this score function is maximized. And hybrid algorithms integrate both constraint-based and score-based approaches.

The algorithms and scores used in structure learning have a certain effect on the determined structure, and different approaches have been developed to improve the BN and the DBN structure learning process. For instance, for the search method used in structure learning, various algorithms including Hill Climbing, Tabu Search, Genetic Algorithm [19], Simulated Annealing [20], Evolutionary Algorithm [21] etc. were included in the structure learning process and their performances were examined. It has been observed that many applications give successful results in the structure learning process.

This study, on the other hand, focused on the performances of the various score criteria on DBN structure learning. In the literature, it has been observed that the most commonly used score functions are K2 [22], Akaike Information Criterion (AIC) [23], Bayesian Information Criterion (BIC) [24], Bayesian Dirichlet Equivalence (BDE) [25] for discrete variables, and AIC-G, BIC-G, Bayesian Gaussian Equivalence (BGE) [26,27] for continuous variables.

The score function has a significant effect on DBN structure learning, and, the objective of this study was to investigate different scores that may be more effective than the scores commonly used in the literature in the DBN structure learning process. For this purpose, alternative scores to the scores commonly preferred in the literature were selected and their effects on DBN structure learning were examined.

Thus, their performance was examined by including eight alternate scoring criteria in the DBN structure learning process. We investigated the performances of the alternate AIC criteria which are KIC [28], AIC4 [29], CAIC [30] and the alternate BIC criteria which are BICadj [31,32], HBIC [33], BICQ [34] in hybrid structure learning in the continuous-state DBN.

The rest of the study was structured as follows. In Material and Method, theoretical information about DBN, the hybrid structure learning process, and the algorithms used in structure learning was provided. Further, information criteria and Vector AutoRegressive (VAR) models were discussed. Experiments, Results and Discussion, and Conclusions were presented in the subsequent sections, with suggestions for future studies included in the conclusion.

## Materials and methods

### Dynamic Bayesian Networks

DBNs are used in modelling multivariate time series or sequential datasets [35]. DBNs are the directed acyclic graphs and they represents the joint probability distribution for a group of random variables X. In DBNs, the variables are called nodes, and the arrows showing the dependencies or causal relationships between the variables are called edges. Precisely, a DBN for *X* is defined as a pair B=(V,E), where *V* represents the set of random variables $X_{\text{1}},X_{\text{2}},\ldots ,X_{n}$, and *E* denotes the set of edges (or arcs) connecting these nodes.

DBNs typically rest on two key assumptions: the first-order Markov property, which asserts that the state of the system at time *t*, denoted as $X_{t}$, is conditionally independent of all earlier states given the immediately preceding state, i.e.,


$$\text{P}\left[X_{t}|X_{t-\text{1}},\ldots ,X_{\text{1}}\right]=P\left(X_{t}|X_{t-\text{1}}\right),$$ (1)

and the stationarity assumption, which posits that the model’s structure and parameters remain constant over time.

Let $P_{B}$ denote the joint probability distribution of the variables over discrete time t∈[1:T], the joint probability function over the entire sequence of variables for a DBN is represented as


$$P_{B}\left[\text{X}(t)\right]=\prod_{t=\text{1}}^{T}\prod_{i=\text{1}}^{n}P_{B}\left[X_{i}(t)|Pa\left(X_{i}(t)\right)\right],$$ (2)

where $Pa\left[X_{i}(t)\right]$ denotes the set of the parents of $X_{i}$ in time slice *t*. With a DBN, both the causality relationships between nodes in the same *t*ime slice (intra-slice arcs) and the dependency structures between nodes in different time slices (inter-slice arcs) can be determined. Thus, the probability distribution for the entire network belonging to the set of variables at different time points can be obtained with the DBNs [36].

### Hybrid structure learning algorithms

The hybrid algorithms are composed of two distinct phases. Initially, constraint-based algorithms are employed. At this stage, all the independence and dependence relationships among variables are established through conditional independence tests, and the skeleton of the network is identified. Frequently used tests for conditional independence include the Pearson correlation test, the likelihood ratio test, the conditional joint knowledge test, and tests for conditional partial relationships. The most widely used constraint-based algorithms are Grow-Shrink (GS) [37], Incremental Association Markov Blanket (IAMB) [38], Inter-Incremental Association Markov Blanket (INTER-IAMB) [39] Fast Incremental Association Markov Blanket (FAST-IAMB) [39] and Min-Max Parents and Children (MMPC) algorithm [40,41], Parent-Children Markov Blanket (PCMB) [42].

In the second step, the score-based algorithms are applied. The score-based algorithms use a search method and a scoring function. Each candidate network structure is given a score that measures its goodness of fit, which the algorithm aims to maximize. Examples of heuristic approaches used to achieve this include greedy search, genetic algorithms, ant colony optimization, and particle swarm optimization [43,44].

The IAMB algorithm utilizes a two-phase method to uncover Markov Blankets under assumptions the existence and faithfulness of a BN, and conditional independence tests are reliable.

**Definition 1 (Markov Blanket)** The Markov blanket of a variable T denoted as MB(T) is the smallest set such that, when conditioned on this set, all other nodes in the network become independent of T.

**Definition 2 (Faithfulness)** A BN B and a joint distribution P are considered faithful to each other if and only if every conditional independence implied by the graph B is also reflected in the distribution P.

Given the faithfulness assumption, MB(T) exists and is unique. During the initial (expansion) phase, all variables that are part of the blanket, along with some potential false positives, are included in MB(T). In the second (shrinking) phase, false positives are identified and removed. The IAMB algorithm employs an information-theoretic heuristic to rank variables during the expansion phase. It rearranges the set of variables each time a new variable is added to the blanket [45].

The Tabu search algorithm, initially introduced by Glover [46], is a local search technique designed to find optimal or near-optimal solutions for optimization problems. This method involves iteratively exploring adjacent solutions through a neighborhood search process.

The algorithm most generally starts with a random initial solution. The algorithm has long-term and short-term memory features. During the search, the algorithm memorizes the best solution it finds with its long-term memory feature and evaluates it by comparing it with other possible solutions.

To prevent cycling between the same solutions, certain moves can be forbidden, and forbidden moves are listed in the tabu list and these movements are not allowed. The tabu list provides the algorithm with a form of short-term memory. While evaluating the neighborhood structures, it is checked whether the move to be made is in the tabu list, and movement designs are generated for different neighborhood structures in the tabu list. According to the length of the determined tabu list, the tabu list has a self-renewal feature.

The Tabu algorithm continues its search until one or more stopping conditions are met. Some of these conditions are that a selected neighbour solution has no neighbours, a certain number of iterations is reached, a certain solution value is reached, or the algorithm is stuck somewhere and cannot produce better results.

### Information criteria

Model selection predominantly relies on information or score criteria, which are primarily grounded in the penalized likelihood function. Model selection based on a criterion entails selecting the model that maximizes the penalized log-likelihood. This is achieved by identifying the highest value of the penalized log-likelihood, where the penalized log-likelihood is derived from the likelihood function of the dataset and a penalty term, which may be a constant or vary depending on the sample size and/or the number of model parameters. The penalty term deals with both model fit and complexity.

Information criteria often differ according to the penalty term they receive. The penalty term may incorporate factors such as the number of parameters in the model, the number of observations in the dataset, or the covariance matrix of the model’s parameters. The most commonly known information criteria are Akaike-based and Bayesian-based information criteria.

AIC [23] and the other criteria linked to AIC are derived from the relative Kullback-Leibler (KL) divergence, a nonparametric measure that assesses the difference between the estimated and the true distributions. AIC seeks to identify the model that minimizes the KL divergence, thus selecting an approximate model that offers improved predictions of the population parameters. Different AIC-based criteria have been developed for different versions of the AIC penalty term and AIC and other AIC-based criteria used in this study were given in Table 1.

**Table 1:** Information criteria used in DBN hybrid structure learning.

Score Criterion	Formula
BIC	−2ln(L)+kln(n)
BICadj	−2ln(L)+kln[(n+2)/24]
HBIC	−2ln(L)+kln[n/(2π)]
BICQ	−2ln(L)+kln(n)−2kln[q/(1−q)]
AIC	−2ln(L)+2k
KIC	−2ln(L)+3k
AIC4	−2ln(L)+4k
CAIC	−2ln(L)+k[ln(n)+1]

BIC information criterion (Schwarz criterion) [24] and the criteria related to BIC [47] are based on the Bayes theorem and deriving a candidate model as an asymptotic approximation to the transformation of the Bayesian posterior probability. It represents the candidate model that is most likely given the posterior distribution. Used BIC and other BIC-based criteria in this study were given in Table 1. In Table 1, L: likelihood function, n: sample size, k: number of parameters, *q*: 0.25.

In line with the implementation of the bnlearn package in R, all information criteria were computed as the log-likelihood minus the complexity penalty. Therefore, larger scores correspond to better-fitting, more parsimonious models. While Table 1 shows the standard definitions (where lower is better), our experiments and tables report scores as implemented, with higher scores indicating better fit.

### VectorAutoRegressive models

In this study, multivariate time series datasets were simulated for structure learning in the analyses and the VAR models were used to generate these datasets. The VAR models are used to model the multivariate time series. The structure involves each variable being a linear function of its own past values as well as the past values of other variables. A lag is the value of a variable in a previous time period. The basic p-order (lag) VAR(*p*) model is of the form


$$x_{t}=A_{\text{1}}x_{t-\text{1}}+\ldots +A_{P}x_{t-p}+\varepsilon_{t},$$ (3)

where $x_{t}=\left(x_{\text{1}t},\ldots ,x_{nt}\right)$ is a (nx1) vector of variables, $A_{i}$ are fixed (nxn) coefficient matrices, $\varepsilon_{t}=\left(\varepsilon_{\text{1}t},\ldots ,\varepsilon_{nt}\right)$ is an (nx1) vector white noise process. The white noise process is a continuous random vector where $E\left(\varepsilon_{t}\right)=\text{0}$, $\sum_{\varepsilon }=E\left(\varepsilon_{t}{\varepsilon_{s}}^{\prime }\right)$ is the non-singular covariance matrix, $\varepsilon_{t}$, and $\varepsilon_{s}$ are independent for s≠t [48].

### Experiments

This study utilized a hybrid method for learning the structure of the continuous-state DBN. The algorithms used in structure learning were given in Table 2.

**Table 2:** The algorithms and score criterion used in DBN hybrid structure learning.

Score-based algorithms	Constraint-based algorithms	Score criteria
TABU	IAMB	AIC-G, KIC, AIC4, CAIC BIC-G, BICadj, HBIC, BICQ

Analyses were performed on three simulated and a real multivariate time series dataset. For datasets, the hybrid structure learning process for the DBN was carried out for each scoring criterion in Table 2. Next, score measure values were computed to assess the performance of the learned network structures. The arithmetic means of these score values, obtained from various sample units after 30 iterations, were then calculated and analyzed.

R program was used for all analyses. The packages used were “bnlearn” [49] and “dbnR” [50] packages for DBN analysis; “BigVAR” [51] for generating simulated datasets.

### Simulated datasets generation

A VAR(1) model was employed to generate the stationary multivariate time series datasets. The variables’ numbers were determined to be 5, 7, and 10, and the number of observations was 1000. Then, structure learning was carried out by taking samples of 100, 500, and 1000 units for each dataset. A VAR(1) model is


$$x_{t}=Cx_{t-\text{1}}+e_{t},$$ (4)

where *C* denotes the coefficient (connectivity) matrix and the coefficient matrices $C_{\text{5}},C_{\text{7}},C_{\text{1}\text{0}}\;$ simulated according to the variable numbers determined as 5,7,10, respectively, were given below.


$$C_{\text{5}}=[\begin{array}{ccccc} \text{0} & -\text{0}.\text{4}\text{7} & -\text{0}.\text{1}\text{1} & \text{0} & \text{0}\\ -\text{0}.\text{9}\text{5} & \text{0} & \text{0} & \text{0} & -\text{0}.\text{4}\text{5}\\ -\text{0}.\text{6}\text{8} & \text{0} & \text{0} & -\text{0}.\text{6}\text{7} & \text{0}.\text{4}\text{8}\\ \text{0} & \text{0} & \text{0}.\text{1}\text{5} & \text{0} & -\text{0}.\text{8}\text{0}\\ -\text{0}.\text{6}\text{3} & \text{0} & \text{0} & \text{0} & \text{0} \end{array}]$$

$$C_{\text{7}}=[\begin{array}{ccccccc} \text{0} & -\text{0}.\text{8}\text{5} & -\text{0}.\text{9}\text{4} & \text{0}.\text{1}\text{6} & \text{0}.\text{7}\text{6} & \text{0} & -\text{0}.\text{2}\text{3}\\ \text{0}.\text{1}\text{5} & \text{0} & \text{0} & \text{0}.\text{2}\text{7} & \text{0} & \text{0} & \text{0}\\ \text{0} & \text{0} & \text{0} & \text{0} & -\text{0}.\text{6}\text{4} & \text{0}.\text{3}\text{9} & -\text{0}.\text{2}\text{8}\\ \text{0} & \text{0} & \text{0} & \text{0}.\text{3}\text{8} & \text{0} & \text{0} & \text{0}.\text{4}\text{8}\\ \text{0} & \text{0}.\text{1}\text{0} & \text{0}.\text{6}\text{4} & \text{0} & \text{0} & \text{0} & \text{0}\\ -\text{0}.\text{6}\text{6} & \text{0}.\text{4}\text{4} & \text{0} & \text{0}.\text{3}\text{8} & \text{0}.\text{5}\text{2} & \text{0} & \text{0}.\text{1}\text{3}\\ \text{0} & \text{0} & \text{0} & \text{0} & -\text{0}.\text{6}\text{5} & \text{0} & -\text{0}.\text{0}\text{5} \end{array}]$$

$$C_{\text{1}\text{0}}=[\begin{array}{cccccccccc} \text{0} & -\text{0}.\text{5}\text{6} & \text{0} & \text{0} & \text{0} & \text{0} & \text{0}.\text{2}\text{9} & -\text{0}.\text{1}\text{1} & \text{0}.\text{7}\text{8} & \text{0}\\ \text{0}.\text{2}\text{1} & \text{0}.\text{3}\text{9} & \text{0} & -\text{0}.\text{5}\text{8} & \text{0} & \text{0} & \text{0} & -\text{0}.\text{2}\text{7} & \text{0}.\text{9}\text{5} & \text{0}.\text{5}\text{4}\\ \text{0} & \text{0}.\text{8}\text{6} & \text{0} & \text{0} & \text{0} & \text{0}.\text{3}\text{3} & \text{0}.\text{5}\text{3} & \text{0} & \text{0} & \text{0}\\ \text{0}.\text{5}\text{8} & \text{0} & \text{0} & -\text{0}.\text{8}\text{9} & \text{0} & \text{0}.\text{7}\text{2} & \text{0} & \text{0} & \text{0} & \text{0}\\ -\text{0}.\text{8}\text{3} & -\text{0}.\text{7}\text{1} & -\text{0}.\text{7}\text{5} & \text{0}.\text{0}\text{2} & \text{0}.\text{1}\text{2} & \text{0} & \text{0}.\text{3}\text{1} & \text{0} & \text{0} & \text{0}\\ -\text{0}.\text{1}\text{3} & \text{0}.\text{2}\text{2} & -\text{0}.\text{0}\text{9} & \text{0} & \text{0} & \text{0} & \text{0} & \text{0} & \text{0} & -\text{0}.\text{4}\text{3}\\ -\text{0}.\text{1}\text{8} & \text{0}.\text{3}\text{9} & \text{0}.\text{8}\text{8} & \text{0} & \text{0} & -\text{0}.\text{5}\text{6} & \text{0} & \text{0}.\text{1} & \text{0}.\text{2}\text{2} & \text{0}.\text{0}\text{8}\\ \text{0}.\text{3}\text{1} & \text{0} & \text{0} & \text{0} & \text{0}.\text{8}\text{1} & \text{0} & \text{0} & \text{0} & \text{0} & \text{0}.\text{3}\text{4}\\ \text{0} & \text{0} & -\text{0}.\text{5}\text{7} & \text{0} & \text{0}.\text{2}\text{8} & \text{0} & \text{0} & \text{0} & \text{0} & -\text{0}.\text{3}\text{4}\\ -\text{0}.\text{9}\text{1} & \text{0} & \text{0} & -\text{0}.\text{3}\text{4} & \text{0}.\text{7}\text{7} & -\text{0}.\text{3}\text{2} & -\text{0}.\text{0}\text{8} & -\text{0}.\text{6}\text{2} & \text{0} & \text{0} \end{array}]$$

The covariance matrices of residuals were determined as diagonal matrices that are the diagonal elements equal to $\sigma^{\text{2}}$(variance) and off-diagonal elements equal to 0. The variances of residuals were generated to be random between 1 and 5.

To verify the stationarity assumption of the simulated VAR(1) processes, we computed the eigenvalues of the coefficient matrices for each dimensionality setting (k = 5, 7, 10). Table 3 the full set of eigenvalues for each k. In all configurations, the maximum modulus was below 1, confirming that the simulated processes are stationary and thus appropriate for DBN structure learning.

**Table 3:** Eigenvalues of the coefficient matrices for the simulated VAR(1) processes used in the experiments.

k	Eigenvalues
5	−0.828, 0.643, 0.005±0.379i, 0.175
7	−0.391±0.864i, 0.547±0.290i, 0.140 ± 0.373i, −0.262
10	−0.273±0.901i, 0.624±0.690i, −0.853±0.254i, −0.865, 0.680±0.224i, 0.128

### UCI datasets

#### Istanbul stock exchange dataset.

Istanbul Stock Exchange (ISE) dataset includes returns of Istanbul Stock Exchange with seven other international index; SP, DAX, FTSE, NIKKEI, BOVESPA, MSCE_EU, MSCI_EM from Jun 5, 2009 to Feb 22, 2011 [52]. The dataset can be accessed in the UCI Machine Learning Repository [53].

In ISE data pre-processing, the previous values were assigned to the missing values and according to the Schwarz information criterion, the optimal lag order was found to be 1. The Augmented Dickey-Fuller (ADF) test was employed to assess the stationarity of the dataset. The results obtained for the ADF unit root test for ISE data were given in Table 4.

**Table 4:** ADF test results for ISE dataset.

Variables	Level
ISE	−16.038
SP	−17.032
DAX	−16.106
FTSE	−16.422
NIKKEI	−13.799
BOVESPA	−16.303
EU	−16.681
EM	−15.675

The null hypothesis posits that the series is non-stationary or contains a unit root. This hypothesis is rejected if the test statistic falls below the MacKinnon critical values of −3.43, −2.86, and −2.56 at the 1%, 5%, and 10% significance levels, respectively. Since the test statistics for all series were much lower (more negative) than these critical values, the null hypothesis was rejected for all of them. Therefore, the series in the ISE dataset was considered stationary.

Since the optimal lag order of the dataset was 1, the number of time slices for DBN structure learning was determined as 2. The structure learning process was carried out according to each criterion by taking samples of 100, 250, and 500 units from the dataset. To assess the performance of the learned network structures, score values were determined using the BIC-G criterion. The arithmetic mean of the score measurements, calculated based on the number of observations, was determined.

#### Air quality dataset.

The Air quality dataset includes 9,358 hourly averaged readings from five metal oxide chemical sensors embedded in an Air quality Chemical Multisensory Device. This device was deployed in a highly polluted area at street level in an Italian city. Data were collected from March 2004 to February 2005 [54]. The dataset can be accessed in the UCI Machine Learning Repository [53].

In Air quality data pre-processing, the previous values were assigned to the missing values tagged as −200. According to the Schwarz information criterion, the optimal lag order was found to be 8. The ADF test was employed to assess the stationarity of the dataset. The results obtained for the ADF unit root test for Air quality data were given in Table 5.

**Table 5:** ADF test results for Air quality dataset.

Variables	Level	First Difference
CO(GT)	−9.423843	−20.085315
PT08.S1(CO)	−9.875915	−10.850750
NMHC(GT)	−10.66215	−10.854134
C6H6(GT)	−10.2559	−20.271827
PT08.S2(NMHC)	−10.17304	−19.927442
NOx(GT)	−7.231727	−26.072267
PT08.S3(NOx)	−10.60454	−18.946013
NO2(GT)	−8.110669	−24.004352
PT08.S4(NO2)	−6.231035	−10.876347
PT08.S5(O3)	−10.78114	−10.818706
T	−3.207005	−21.721202
RH	−7.317636	−20.235526
AH	−5.094239	−19.963247

The null hypothesis posits that the series is non-stationary or contains a unit root. This hypothesis is rejected if the test statistic falls below the critical values. The MacKinnon critical values for the ADF test were −3.43, −2.86, and −2.56 for %1, %5, and %10 levels, respectively. The results in Table 5 indicated that the first difference of each series was stationary.

Consequently, the first differenced Air quality data was used for DBN structure learning. Since the optimal lag order of the dataset was 8, the number of time slices for DBN structure learning was determined as 9. The structure learning process was carried out according to each criterion by taking samples of 100, 500, and 1000 units from the dataset. To assess the performance of the learned network structures, score values were determined using the BIC-G criterion. The arithmetic mean of the score measurements, calculated based on the number of observations, was determined.

## Results and discussion

### Simulated datasets

#### Score measurements for simulated datasets.

In this section, we presented the analysis results. DBN structure learning was performed for all Datasets with the combination of algorithms and score criteria in Table 2. To evaluate the performance of the learned network structures, we generated 30 independent simulated datasets for each configuration, each consisting of 1000 observations, and applied the structure learning procedure separately to each dataset. In each replication, an independent time-series dataset of 1000 observations was simulated using the same underlying parameters (coefficient matrix and covariance matrix). To assess the impact of sample size, three different subsets of the simulated data were extracted: the first 100, 500, and 1000 observations, respectively. DBN structure learning was then performed for each of these subsets using the combinations of algorithms and score criteria listed in Table 2. For each criterion and setting, scores and SHD values were computed in each replication. Finally, the arithmetic mean, and standard error (SE) of the scores were calculated. These results were reported in Table 6 for simulated datasets to facilitate comparison across different dimensional settings.

**Table 6:** The mean scores and SE for the simulated datasets (Higher (less negative) scores indicate better model fit).

		n = 100		n = 500		n = 1000	
k	Criterion	Mean	SE	Mean	SE	Mean	SE
5	AIC-G	−2331.916	8.732	−11530.301	16.585	−22986.160	19.198
	KIC	−2331.916	8.732	−11530.301	16.585	−22986.160	19.198
	AIC4	−2331.851	8.730	−11530.244	16.581	−22986.160	19.198
	CAIC	−2331.529	8.744	−11529.194	16.545	−22984.978	19.214
	BIC-G	−2331.635	8.727	−11529.446	16.551	−22984.988	19.229
	BICadj	−2331.916	8.732	−11530.301	16.585	−22986.160	19.198
	HBIC	−2331.916	8.732	−11530.077	16.592	−22985.804	19.172
	BICQ	−2331.117	8.800	−11528.785	16.535	−22984.371	19.216
7	AIC-G	−3319.585	15.181	−16232.410	46.073	−32248.931	69.534
	KIC	−3319.585	15.181	−16232.410	46.073	−32248.931	69.534
	AIC4	−3319.585	15.181	−16234.518	45.485	−32250.260	69.705
	CAIC	−3319.626	15.196	−16234.473	45.458	−32249.757	69.584
	BIC-G	−3319.642	15.180	−16234.570	45.462	−32249.918	69.614
	BICadj	−3319.585	15.181	−16232.410	46.073	−32250.260	69.705
	HBIC	−3319.585	15.181	−16234.533	45.482	−32249.937	69.691
	BICQ	−3319.919	15.202	−16237.791	45.080	−32250.085	69.494
10	AIC-G	−5623.824	25.395	−26678.958	54.174	−52402.062	73.297
	KIC	−5623.824	25.395	−26678.958	54.174	−52402.056	73.297
	AIC4	−5623.824	25.395	−26678.958	54.174	−52398.157	73.688
	CAIC	−5626.304	24.675	−26678.958	54.174	−52402.885	74.312
	BIC-G	−5625.202	24.751	−26678.958	54.174	−52403.103	74.334
	BICadj	−5623.824	25.395	−26678.958	54.174	−52402.056	73.297
	HBIC	−5623.824	25.395	−26678.958	54.174	−52398.713	73.669
	BICQ	−5627.593	24.563	−26678.958	54.174	−52397.280	76.005

Table 6 summarizes the mean scores and their SE over 30 replications for each scoring criterion across datasets with 5, 7, and 10 variables and varying sample sizes (100, 500, and 1000). Higher (less negative) score values indicate better model fit under the scoring conventions applied in this study.

When the results were compared only in terms of alternative AIC criteria, for the simulated datasets generated with a variable number of 5, the highest measurement results were obtained with CAIC when the number of observations was 100, 500, and 1000. For the simulated datasets generated with a variable number of 7, the highest measurement results were obtained with AIC-G, CAIC, and KIC when the number of observations was 100, with AIC-G and KIC when the number of observations was 500 and 1000. For the simulated datasets generated with a variable number of 10, the highest measurement (least negative) results were obtained with AIC-G, KIC, and CAIC when the number of observations was 100. No difference was observed between the score values for the number of observations of 500. When the number of observations was 1000, the highest measurement results were obtained with AIC4.

When the results were compared only in terms of alternative BIC criteria, while the number of observations was 100, 500, and 1000 for the simulated data generated as the variable number of 5, the highest measurement results were obtained with BICQ. While the number of observations was 100 for the simulated data generated as 7 variables, the highest measurement values were obtained with BICadj and HBIC, with BICadj when the number of observations was 500, and with BIC-G when the number of observations was 1000. For the simulated datasets generated with a variable number of 10, the highest measurement results were obtained with BICadj and HBIC while the number of observations was 100. No difference was observed between the score values for the number of observations of 500. The highest results for the number of observations 1000 and the number of variables 10 were obtained with BICQ.

Across all configurations of variable dimension (k = 5, 7, 10) and sample sizes (n = 100, 500, 1000), the SE of the score criteria remain relatively low compared to the magnitude of the mean scores. This indicates that the structure learning results are stable and reproducible across the 30 independent replications. Furthermore, the SEs are fairly similar across different scoring criteria within each configuration, suggesting that no single criterion introduces substantially more variability than others. Therefore, the observed differences in mean scores can be considered meaningful within the bounds of Monte Carlo variability, although small numerical differences (less than 5–10 units) should still be interpreted with caution given the magnitude of the SEs.

Since the BICQ criterion can be sensitive to the choice of the quantile parameter *q*, we performed a sensitivity analysis by computing the BICQ score under four different *q* values: *q* = 0.25, *q* = 0.75, *q* = 0.85, and *q* = 0.95. *q* = 0.50 was not included in this comparison, as it coincides with the standard BIC when *q* = 0.50 [34]. The analysis was carried out separately for the simulated datasets with k = 5, 7, and 10 variables, each with n = 100, 500, and 1000 observations. For each configuration, the BICQ score were computed to assess the stability and comparative performance of BICQ at different *q* settings. The results obtained were given in Table 7.

**Table 7:** The mean BICQ scores for different *q* values (Higher (less negative) scores indicate better model fit).

		n = 100		n = 500		n = 1000	
k	Criterion	Mean	SE	Mean	SE	Mean	SE
5	q = 0.25	−2315.436	7.852606	−11498.65	14.74537	−22959.18	17.03313
	q = 0.75	−2316.226	7.933536	−11502.05	14.76635	−22961.02	16.97701
	q = 0.85	−2316.226	7.933536	−11502.25	14.74263	−22961.32	16.95216
	q = 0.95	−2316.226	7.933536	−11502.25	14.74263	−22961.32	16.95216
7	q = 0.25	−3290.882	13.08529	16149.94	41.55658	−32167.67	77.21587
	q = 0.75	−3291.948	13.42792	−16149.79	41.51166	−32158.32	76.45111
	q = 0.85	−3291.948	13.42792	−16153.34	41.56431	−32157.51	76.48447
	q = 0.95	−3291.948	13.42792	−16153.34	41.56431	−32157.68	76.52730
10	q = 0.25	−5627.593	24.56304	−26678.96	54.17427	−52397.28	76.00491
	q = 0.75	−5623.824	25.39470	−26678.96	54.17427	−52398.71	73.66875
	q = 0.85	−5623.824	25.39470	−26678.96	54.17427	−52402.06	73.29701
	q = 0.95	−5623.970	25.39222	−26678.13	54.25267	−52402.06	73.29672

Across all scenarios (k = 5, 7, 10) and sample sizes (n = 100, 500, 1000), the mean BICQ scores generally showed only minor changes as *q* increases from 0.25 to 0.95. The differences are small — often less than one unit and within the standard error margins — indicating that the choice of *q* has little effect on scoring performance or the stability of the learned structures. For this reason, *q* = 25 was determined for the BICQ in the study.

#### SHD measurements for simulated datasets.

Furthermore, the SHD values were calculated for the simulated datasets. It gives the criterion of similarity between the original network structure and the predicted network structure of the dataset, and the smaller the SHD value, the more similar the predicted structure to the real network structure.

To evaluate the structural accuracy of the learned DBN models, we computed the SHD between the true underlying network structure and each estimated structure. The true network structure for each scenario (k = 5, 7, 10) was defined a priori based on the VAR(1) coefficient matrices used in data generation, and encoded in the standard model string representation of a BN. At each replication, after learning the DBN structure using a given scoring criterion, the estimated network was compared to the true network using the shd() function from the bnlearn R package. This process was repeated across all 30 independent replications for each configuration and scoring criterion. The resulting SHD values were then summarized by computing their arithmetic mean to assess the variability and consistency of the structure learning performance. The results were given in Table 8.

**Table 8:** The SHD results for simulated datasets (Smaller values indicate better model fit).

	k = 5			k = 7			k = 10		
Criterion	n = 100	n = 500	n = 1000	n = 100	n = 500	n = 1000	n = 100	n = 500	n = 1000
AIC-G	7.16667	12.23333	14.56667	23.1	25.2	25.76667	45.8	43.7	44.46667
KIC	7.16667	12.23333	14.56667	23.1	25.2	25.76667	45.8	43.7	44.46667
AIC4	7.1	12.06667	14.56667	23.1	25.2	25.76667	45.8	43.7	44.46667
CAIC	6.26667	11	13.53333	23.03333	25.1	25.33333	45.53333	43.7	44.5
BIC-G	6.73333	11.23333	13.76667	23.06667	25.16667	25.46667	45.56667	43.7	44.5
BICadj	7.16667	12.23333	14.56667	23.1	25.2	25.76667	45.8	43.7	44.46667
HBIC	7.16667	12	14.33333	23.1	25.2	25.63333	45.8	43.7	44.5
BICQ	5.16667	10.7	13.3	23.06667	25.03333	25.23333	45.7	43.7	44.53333

Table 8 reports the mean SHD values over 30 independent replications for each scoring criterion, across simulated datasets with 5, 7, and 10 variables and varying sample sizes (100, 500, and 1000). Lower SHD values indicate that the learned network structures are closer to the true underlying structure, thus reflecting better structural accuracy.

For the datasets with 5 variables, the lowest SHD values were consistently achieved by BICQ, followed by CAIC and BIC-G. In particular, for sample sizes of 100, 500, and 1000, BICQ yielded mean SHD values of 5.16667, 10.7, and 13.3, respectively, which were noticeably lower than those of other criteria. This suggested that BICQ is more effective at recovering the true network structure in lower-dimensional settings. For the datasets with 7 variables, differences among criteria became less pronounced. All criteria yielded similar SHD values, around 23–25 across sample sizes. For the datasets with 10 variables, SHD values were higher overall—as expected due to increased model complexity—but again fairly consistent across criteria, ranging between 43.7 and 45.8.

Overall, these results indicate that while the impact of the scoring criterion on SHD diminishes as dimensionality increases, BICQ performs better in low-dimensional settings, producing learned structures that are closer to the true model. Nonetheless, in higher-dimensional scenarios, all criteria exhibit comparable performance in terms of SHD, likely reflecting the inherent difficulty of accurately recovering large, complex networks.

In addition to reporting mean SHD values across 30 independent replicates, repeated-measures Analysis fo Variances (ANOVAs) were also performed to assess whether the observed differences between scoring criteria were statistically significant. The analysis were performed using Statiscial Program for Social Sciences (SPSS).

Specifically, for each simulated dataset configuration (5, 7, and 10 variables), we treated the 30 replicates as within-subject observations and the different scoring criteria as repeated measures, and a repeated-measures ANOVA was performed. This approach allowed us to formally test whether the mean SHD differed significantly across scoring criteria within each experimental condition.

For the simulated dataset with 5 variables, SHD values were calculated separately for each of 8 scoring criteria over 30 independent replications for each sample size configuration (n = 100, 500, 1000). Subsequently, for each criterion and each sample size, the mean SHD value across the 30 replications was computed. These mean SHD values were then used as input to a repeated-measures ANOVA to statistically assess whether the observed differences among the scoring criteria were significant.

Prior to the ANOVA, the assumption of normality for the mean SHD values was tested. The results of the normality tests were reported in Table 9 for the first dataset configuration.

**Table 9:** Test of normality results for the dataset with k = 5.

Tests of Normality						
	Kolmogorov-Smirnov^a^			Shapiro-Wilk		
	Statistic	df	Sig.	Statistic	df	Sig.
AICG	,124	30	,200^*^	,972	30	,598
KIC	,124	30	,200^*^	,972	30	,598
AIC4	,143	30	,121	,966	30	,444
CAIC	,108	30	,200^*^	,976	30	,718
BICG	,128	30	,200^*^	,964	30	,381
BICADJ	,124	30	,200^*^	,972	30	,598
HBIC	,128	30	,200^*^	,981	30	,843
BICQ	,108	30	,200^*^	,964	30	,379

*. This is a lower bound of the true significance.

a. Lilliefors Significance Correction

According to the normality test results obtained in Table 7, since the number of observations was 30 < 50, the Sig. (p) values of the Shapiro-Wilk test were examined. Since all scoring criteria p values were greater than 0.05 (Significance level−α), it was concluded that the assumption of a normal distribution of the data was met.

Since the data met the normality assumption, a repeated-measures ANOVA test was applied. The results of the analysis were given in Table 10.

**Table 10:** The repeated-measure ANOVA results for the dataset with k = 5.

Tests of Within-Subjects Effects							
Measure: SHD_Measures							
Source		Type III Sum of Squares	df	Mean Square	F	Sig.	Partial Eta Squared
Scores	Sphericity Assumed	78,251	7	11,179	47,181	,000	,619
	Greenhouse-Geisser	78,251	1,962	39,876	47,181	,000	,619
	Huynh-Feldt	78,251	2,103	37,203	47,181	,000	,619
	Lower-bound	78,251	1,000	78,251	47,181	,000	,619
Error(Scores)	Sphericity Assumed	48,097	203	,237			
	Greenhouse-Geisser	48,097	56,908	,845			
	Huynh-Feldt	48,097	60,998	,789			
	Lower-bound	48,097	29,000	1,659			

In Table 10, it was observed that there was a significant difference between the measurement results since the Sig. of the Greenhouse-Geisser test was 0 (p) < 0.05 (α).

The ANOVA test for repeated-measures for simulated data with 5 variables revealed that the mean SHD values differed according to the score criterion. A pairwise comparison table is provided in Table 11 to determine which score criteria accounted for the differences.

**Table 11:** Pairwise comparisons table for the dataset with k = 5.

Pairwise Comparisons						
Measure: SHD_Measures						
(I) Scores	(J) Scores	Mean Difference (I-J)	Std. Error	Sig.^b^	95% Confidence Interval for Difference^b^	
					Lower Bound	Upper Bound
1	2	,000	,000	.	,000	,000
	3	,078	,059	1,000	-,126	,281
	4	1,055^*^	,163	,000	,495	1,616
	5	,744^*^	,129	,000	,302	1,187
	6	,000	,000	.	,000	,000
	7	,156	,081	1,000	-,123	,434
	8	1,600^*^	,185	,000	,963	2,237
2	1	,000	,000	.	,000	,000
	3	,078	,059	1,000	-,126	,281
	4	1,055^*^	,163	,000	,495	1,616
	5	,744^*^	,129	,000	,302	1,187
	6	,000	,000	.	,000	,000
	7	,156	,081	1,000	-,123	,434
	8	1,600^*^	,185	,000	,963	2,237
3	1	-,078	,059	1,000	-,281	,126
	2	-,078	,059	1,000	-,281	,126
	4	,978^*^	,157	,000	,439	1,516
	5	,667^*^	,119	,000	,259	1,074
	6	-,078	,059	1,000	-,281	,126
	7	,078	,067	1,000	-,153	,309
	8	1,522^*^	,186	,000	,882	2,163
4	1	−1,055^*^	,163	,000	−1,616	-,495
	2	−1,055^*^	,163	,000	−1,616	-,495
	3	-,978^*^	,157	,000	−1,516	-,439
	5	-,311^*^	,086	,031	-,607	-,015
	6	−1,055^*^	,163	,000	−1,616	-,495
	7	-,900^*^	,147	,000	−1,404	-,396
	8	,545^*^	,121	,003	,128	,961
5	1	-,744^*^	,129	,000	−1,187	-,302
	2	-,744^*^	,129	,000	−1,187	-,302
	3	-,667^*^	,119	,000	−1,074	-,259
	4	,311^*^	,086	,031	,015	,607
	6	-,744^*^	,129	,000	−1,187	-,302
	7	-,589^*^	,112	,000	-,972	-,205
	8	,856^*^	,127	,000	,420	1,291
6	1	,000	,000	.	,000	,000
	2	,000	,000	.	,000	,000
	3	,078	,059	1,000	-,126	,281
	4	1,055^*^	,163	,000	,495	1,616
	5	,744^*^	,129	,000	,302	1,187
	7	,156	,081	1,000	-,123	,434
	8	1,600^*^	,185	,000	,963	2,237
7	1	-,156	,081	1,000	-,434	,123
	2	-,156	,081	1,000	-,434	,123
	3	-,078	,067	1,000	-,309	,153
	4	,900^*^	,147	,000	,396	1,404
	5	,589^*^	,112	,000	,205	,972
	6	-,156	,081	1,000	-,434	,123
	8	1,444^*^	,173	,000	,849	2,040
8	1	−1,600^*^	,185	,000	−2,237	-,963
	2	−1,600^*^	,185	,000	−2,237	-,963
	3	−1,522^*^	,186	,000	−2,163	-,882
	4	-,545^*^	,121	,003	-,961	-,128
	5	-,856^*^	,127	,000	−1,291	-,420
	6	−1,600^*^	,185	,000	−2,237	-,963
	7	−1,444^*^	,173	,000	−2,040	-,849

Based on estimated marginal means.

*. The mean difference is significant at the.05 level.

^b^ Adjustment for multiple comparisons: Bonferroni.

Table 11 reported the pairwise comparisons of SHD means across the 8 scoring criteria for the dataset with k = 5, based on repeated-measures ANOVA. The results indicated that several criteria yielded statistically significant differences in SHD performance at the 0.05 significance level. Specifically, CAIC, BIC-G, BICadj, HBIC, and BICQ produced significantly different SHD means compared to AIC-G. Notably, BICQ yielded the lowest SHD among all criteria and was significantly different from most others.

Second, for the 7 variable simulated dataset, SHD values were calculated separately for each of 8 scoring criteria across 30 independent replicates for each sample size configuration. The mean SHD value was then calculated for each criterion. These mean SHD values were used as input to a repeated-measures ANOVA to statistically assess whether the observed differences between the scoring criteria were significant. The results obtained for the normality assumption were presented in Table 12.

**Table 12:** Test of normality results for the dataset with k = 7.

Tests of Normality						
	Kolmogorov-Smirnov^a^			Shapiro-Wilk		
	Statistic	df	Sig.	Statistic	df	Sig.
AICG	,118	30	,200^*^	,950	30	,174
KIC	,118	30	,200^*^	,950	30	,174
AIC4	,118	30	,200^*^	,950	30	,174
CAIC	,154	30	,066	,951	30	,179
BICG	,154	30	,067	,942	30	,105
BICADJ	,118	30	,200^*^	,950	30	,174
HBIC	,127	30	,200^*^	,954	30	,213
BICQ	,119	30	,200^*^	,956	30	,249

*. This is a lower bound of the true significance.

^a^ Lilliefors Significance Correction

Table 12 showed that the normality assumption was met (p > 0.05). Since the normality assumption was met, a repeated measures ANOVA test was performed for the simulated data with 7 variables. The results of the test were given in Table 13.

**Table 13:** The repeated-measure ANOVA results for the dataset with k = 7.

Tests of Within-Subjects Effects							
Measure: SHD_Measures							
Source		Type III Sum of Squares	df	Mean Square	F	Sig.	Partial Eta Squared
Scores	Sphericity Assumed	2,099	7	,300	4,549	,000	,136
	Greenhouse-Geisser	2,099	1,685	1,246	4,549	,020	,136
	Huynh-Feldt	2,099	1,778	1,181	4,549	,018	,136
	Lower-bound	2,099	1,000	2,099	4,549	,042	,136
Error(Scores)	Sphericity Assumed	13,384	203	,066			
	Greenhouse-Geisser	13,384	48,876	,274			
	Huynh-Feldt	13,384	51,558	,260			
	Lower-bound	13,384	29,000	,462			

In Table 13, it was observed that there was a significant difference between the measurement results since the Sig. of the Greenhouse-Geisser test was 0.02 (p) < 0.05 (α).

The ANOVA test for repeated-measures for simulated data with 7 variables revealed that the mean SHD values differed according to the score criterion. A pairwise comparison table was provided in Table 14 to determine which score criteria accounted for the differences.

**Table 14:** Pairwise comparisons table for the dataset with k = 7.

Pairwise Comparisons						
Measure: SHD_Measures						
(I) Scores	(J) Scores	Mean Difference (I-J)	Std. Error	Sig.^a^	95% Confidence Interval for Difference^a^	
					Lower Bound	Upper Bound
1	2	,000	,000	.	,000	,000
	3	,000	,000	.	,000	,000
	4	,200	,088	,878	-,104	,504
	5	,122	,065	1,000	-,101	,345
	6	,000	,000	.	,000	,000
	7	,044	,035	1,000	-,075	,164
	8	,244	,100	,574	-,099	,587
2	1	,000	,000	.	,000	,000
	3	,000	,000	.	,000	,000
	4	,200	,088	,878	-,104	,504
	5	,122	,065	1,000	-,101	,345
	6	,000	,000	.	,000	,000
	7	,044	,035	1,000	-,075	,164
	8	,244	,100	,574	-,099	,587
3	1	,000	,000	.	,000	,000
	2	,000	,000	.	,000	,000
	4	,200	,088	,878	-,104	,504
	5	,122	,065	1,000	-,101	,345
	6	,000	,000	.	,000	,000
	7	,044	,035	1,000	-,075	,164
	8	,244	,100	,574	-,099	,587
4	1	-,200	,088	,878	-,504	,104
	2	-,200	,088	,878	-,504	,104
	3	-,200	,088	,878	-,504	,104
	5	-,078	,059	1,000	-,281	,126
	6	-,200	,088	,878	-,504	,104
	7	-,155	,081	1,000	-,434	,123
	8	,044	,050	1,000	-,127	,216
5	1	-,122	,065	1,000	-,345	,101
	2	-,122	,065	1,000	-,345	,101
	3	-,122	,065	1,000	-,345	,101
	4	,078	,059	1,000	-,126	,281
	6	-,122	,065	1,000	-,345	,101
	7	-,078	,057	1,000	-,274	,118
	8	,122	,079	1,000	-,150	,394
6	1	,000	,000	.	,000	,000
	2	,000	,000	.	,000	,000
	3	,000	,000	.	,000	,000
	4	,200	,088	,878	-,104	,504
	5	,122	,065	1,000	-,101	,345
	7	,044	,035	1,000	-,075	,164
	8	,244	,100	,574	-,099	,587
7	1	-,044	,035	1,000	-,164	,075
	2	-,044	,035	1,000	-,164	,075
	3	-,044	,035	1,000	-,164	,075
	4	,155	,081	1,000	-,123	,434
	5	,078	,057	1,000	-,118	,274
	6	-,044	,035	1,000	-,164	,075
	8	,200	,091	1,000	-,114	,514
8	1	-,244	,100	,574	-,587	,099
	2	-,244	,100	,574	-,587	,099
	3	-,244	,100	,574	-,587	,099
	4	-,044	,050	1,000	-,216	,127
	5	-,122	,079	1,000	-,394	,150
	6	-,244	,100	,574	-,587	,099
	7	-,200	,091	1,000	-,514	,114

Based on estimated marginal means.

^a^ Adjustment for multiple comparisons: Bonferroni.

Pairwise comparisons in Table 14 revealed that no pairwise differences between the scoring criteria were statistically significant (all p > 0.05). This finding suggests that, despite the repeated-measures ANOVA analysis showing a generally significant difference between the criteria, it was not clear which two criteria accounted for these differences. In other words, while ANOVA indicates that overall variation is dependent on the criteria, pairwise comparisons revealed that these differences did not reach statistical significance. This could be interpreted as indicating that the scoring criteria performed similarly and that the differences were small.

Thirdly, the results of the repeated measures ANOVA test for the simulated data with 10 variables were given in Tables 15-16.

**Table 15:** Test of normality results for the dataset with k = 10.

Tests of Normality						
	Kolmogorov-Smirnov^a^			Shapiro-Wilk		
	Statistic	df	Sig.	Statistic	df	Sig.
AICG	,114	30	,200^*^	,965	30	,406
KIC	,114	30	,200^*^	,965	30	,406
AIC4	,114	30	,200^*^	,965	30	,406
CAIC	,099	30	,200^*^	,942	30	,102
BICG	,100	30	,200^*^	,960	30	,304
BICADJ	,114	30	,200^*^	,965	30	,406
HBIC	,117	30	,200^*^	,955	30	,226
BICQ	,115	30	,200^*^	,949	30	,161

*. This is a lower bound of the true significance.

^a^ Lilliefors Significance Correction

**Table 16:** The repeated-measure ANOVA results for the dataset with k = 10.

Tests of Within-Subjects Effects							
Measure: SHD_Measures							
Source		Type III Sum of Squares	df	Mean Square	F	Sig.	Partial Eta Squared
Scores	Sphericity Assumed	,244	7	,035	,925	,488	,031
	Greenhouse-Geisser	,244	1,391	,175	,925	,373	,031
	Huynh-Feldt	,244	1,439	,170	,925	,376	,031
	Lower-bound	,244	1,000	,244	,925	,344	,031
Error(Scores)	Sphericity Assumed	7,654	203	,038			
	Greenhouse-Geisser	7,654	40,328	,190			
	Huynh-Feldt	7,654	41,719	,183			
	Lower-bound	7,654	29,000	,264			

The dataset with the number of variables k = 10 provided the normality assumption (all p > 0.05).

The repeated-measures ANOVA on SHD measures in Table 15 revealed no statistically significant differences among the scoring criteria (p = 0.373 > 0.05=α). The effect size was small, indicating that the choice of scoring criterion did not significantly impact the SHD values in this dataset.

### UCI datasets

In this section, DBN structure learning analyses were performed to compare different scoring functions on the ISE and Air quality datasets. Samples were randomly selected 30 times for each sample size. The DBN structure was learned using a total of 8 different scoring functions on each sample: AIC-G, KIC, AIC4, CAIC, BIC-G, BICadj, HBIC, and BICQ. The fitness of the learned networks was evaluated, and the mean and the standard error values for each scoring criterion were reported. The results of UCI datasets were given in Tables 17 and 18, respectively.

**Table 17:** The score measurement results for the ISE dataset (Higher scores indicate better model fit).

	n = 100		n = 250		n = 500	
Criterion	Mean	SE	Mean	SE	Mean	SE
AIC-G	5206.440	50.54419	13122.83	113.2705	26458.48	10.2233716
KIC	5235.866	58.11729	13123.58	114.0061	26448.28	0.9656414
AIC4	5201.776	81.52516	13123.58	114.0061	26448.28	0.9656414
CAIC	5201.351	81.94973	13164.51	184.2961	26448.51	NA
BIC-G	5201.776	81.52516	13164.51	184.2961	26448.18	1.3588947
BICadj	5189.065	42.83372	13123.58	114.0061	26448.28	0.9656414
HBIC	5206.440	50.54419	13123.58	114.0061	26448.18	1.3588947
BICQ	5283.301	NA	13346.61	NA	26448.51	NA

**Table 18:** The score measurement results for the Air quality dataset (Higher (less negative) scores indicate better model fit).

	n = 100		n = 500		n = 1000	
Criterion	Mean	SE	Mean	SE	Mean	SE
AIC-G	−38326.60	174.2228	−202766.7	503.4879	−409325.1	616.7329
KIC	−38326.44	174.2231	−202766.1	503.5568	−409495.6	613.2524
AIC4	−38326.40	174.2549	−202770.0	502.0271	−409426.1	642.2453
CAIC	−38286.39	175.2987	−202365.6	607.2682	−409558.0	805.6852
BIC-G	−38326.44	174.2507	−202536.7	503.6046	−409719.2	727.2276
BICadj	−38326.92	174.2137	−202766.0	503.5713	−409414.9	640.6610
HBIC	−38326.50	174.2253	−202685.2	537.4487	−409414.7	676.0873
BICQ	−38288.36	175.3504	−202366.0	607.2512	−409465.4	936.6359

According to the results in the Table 17, BICQ generally provides the highest average score for small (n = 100) and medium (n = 250) samples. It outperforms the other criteria, with an average score of 5283.301 for a sample size of n = 100 and 13346.61 for a sample size of n = 250. This suggests that BICQ may be more advantageous for small and medium-sized data.

As the sample size increases, reaching n = 500, the differences between the criteria appear to diminish significantly. At this point, AIC-G provides a slightly higher score; in other words, the performance of the classical criteria and BICQ becomes quite similar on large datasets.

The SE values also provide an important clue. Uncertainty is higher for some criteria, particularly in small sample sizes but SE values decrease significantly as n = 250 and n = 500 increase. This demonstrates that the scores obtained become more consistent and reliable as the sample size increases.

In Table 17, SE values for some criteria could not be calculated and were reported as NA. This is believed to be due to the variance being close to zero due to the very similar scores obtained across replicates, and is methodologically expected. However, caution should be exercised when assessing the uncertainty level of these criteria.

In Table 18, when the score measurements were examined only in terms of alternate AIC scores, the highest score measurements were obtained with CAIC for the number of observations 100 and 500 respectively, and with the AIC-G score the number of observations 1000. When the score measurements were examined only in terms of alternate BIC scores, the highest score measurements were obtained with BICQ for the number of observations 100 and 500 respectively, and with the BICadj and HBIC score the number of observations 1000. When the results were evaluated in terms of all score criteria, it was seen that the BICQ score stands out for small and medium samples (n = 100, 500).

## Conclusions

In this study, the performances of score criteria on the hybrid structure learning process were examined by including the AIC-based score criteria (AIC-G, KIC, AIC4, CAIC), and the BIC-based score criteria (BIC-G, BICadj, HBIC, BICQ) in addition to the scores commonly used in the literature for the score criteria used in the structure learning process in DBN.

It was concluded that the network structure estimates, which are more compatible with the data set, differ according to the data set and score criteria. However, the analysis revealed that the BICQ score generally stood out, exhibiting strong performance on smaller data sets. It is thought that this study will be a starting point for researchers in similar fields and contribute to their studies.

In this study, we fixed the hybrid strategy to IAMB in the restriction phase and Tabu Search in the score-based phase to isolate and clearly assess the impact of the scoring criteria. We emphasize that the findings reported here pertain specifically to this chosen combination, and future studies could investigate whether similar patterns hold for alternative hybrid algorithms.

While our experiments focused on moderate-dimensional settings with up to 10 variables and sufficient sample sizes, we acknowledge that many modern DBN applications often involve much higher dimensions and limited samples. Exploring how our findings generalize to such high-dimensional, small-sample contexts remains an important direction for future work.

In future studies, the performance of the BN and/or DBN structure learning process can be examined by investigating different score criteria other than the scores used in this study. This study focused only on the effects of score criteria in the structure learning process. However, there are factors such as search methods, constraint-based algorithms, and independence tests as well as scoring criteria used in the structure learning process. By offering alternatives in these areas, studies that will contribute to the development of the DBN structure learning process can be developed.

## References

[1] Uusitalo L. Advantages and challenges of Bayesian networks in environmental modelling. Ecological Modelling. 2007;203(3–4):312–8. doi: 10.1016/j.ecolmodel.2006.11.033

[2] Castillo E, Menéndez JM, Sánchez-Cambronero S. Predicting traffic flow using Bayesian networks. Transportation Research Part B: Methodological. 2008;42(5):482–509. doi: 10.1016/j.trb.2007.10.003

[3] Mascaro S, Nicholso AE, Korb KB. Anomaly detection in vessel tracks using Bayesian networks. International Journal of Approximate Reasoning. 2014;55(1):84–98. doi: 10.1016/j.ijar.2013.03.012

[4] Przytula KW, Thompson D. Construction of Bayesian networks for diagnostics. In: 2000 IEEE Aerospace Conference. Proceedings (Cat. No.00TH8484). 193–200. doi: 10.1109/aero.2000.878490

[5] Trovati M, Hayes J, Palmieri F, Bessis N. Automated extraction of fragments of Bayesian networks from textual sources. Applied Soft Computing. 2017;60:508–19. doi: 10.1016/j.asoc.2017.07.009

[6] Darwich A. Modeling and reasoning with Bayesian networks. Cambridge University Press. 2009.

[7] Guo H, Liu X, Sun Z. Multivariate time series prediction using a hybridization of VARMA models and Bayesian networks. Journal of Applied Statistics. 2016;43(16):2897–909. doi: 10.1080/02664763.2016.1155111

[8] Pearl J. Decision making under uncertainty. ACM Comput Surv. 1996;28(1):89–92. doi: 10.1145/234313.234354

[9] Constantinou AC, Fenton N. Things to know about Bayesian Networks: Decisions under Uncertainty, Part 2. Significance. 2018;15(2):19–23. doi: 10.1111/j.1740-9713.2018.01126.x

[10] Scanagatta M, Salmerón A, Stella F. A survey on Bayesian network structure learning from data. Prog Artif Intell. 2019;8(4):425–39. doi: 10.1007/s13748-019-00194-y

[11] Marcot BG, Penman TD. Advances in Bayesian network modelling: Integration of modelling technologies. Environmental Modelling & Software. 2019;111:386–93. doi: 10.1016/j.envsoft.2018.09.016

[12] Sevinc V, Kucuk O, Goltas M. A Bayesian network model for prediction and analysis of possible forest fire causes. Forest Ecology and Management. 2020;457:117723. doi: 10.1016/j.foreco.2019.117723

[13] Amin MdT, Khan F, Ahmed S, Imtiaz S. A data-driven Bayesian network learning method for process fault diagnosis. Process Safety and Environmental Protection. 2021;150:110–22. doi: 10.1016/j.psep.2021.04.004

[14] Yuan Z, Zhuo K, Zhang Q, Zhao C, Sang S. Probabilistic assessment of visual fatigue caused by stereoscopy using dynamic Bayesian networks. Acta Ophthalmol. 2019;97(3):e435–41. doi: 10.1111/aos.13784 29696801

[15] Zhang Z, Dong F. Fault detection and diagnosis for missing data systems with a three time-slice dynamic Bayesian network approach. Chemometrics and Intelligent Laboratory Systems. 2014;138:30–40. doi: 10.1016/j.chemolab.2014.07.009

[16] Chen J, Zhong P-A, An R, Zhu F, Xu B. Risk analysis for real-time flood control operation of a multi-reservoir system using a dynamic Bayesian network. Environmental Modelling & Software. 2019;111:409–20. doi: 10.1016/j.envsoft.2018.10.007

[17] Kim SY, Imoto S, Miyano S. Inferring gene networks from time series microarray data using dynamic Bayesian networks. Brief Bioinform. 2003;4(3):228–35. doi: 10.1093/bib/4.3.228 14582517

[18] Tucker A, Liu X. Learning dynamic Bayesian networks from multivariate time series with changing dependencies. In: International Symposium on Intelligent Data Analysis, Berlin, Heidelberg, 2003. 100–10.

[19] Larranaga P, Sierra B, Gallego MJ, Michelena MJ, Picaza JM. Learning Bayesian networks by genetic algorithms: a case study in the prediction of survival in malignant skin melanoma. In: Artificial Intelligence in Medicine: 6th Conference on Artificial Intelligence in Medicine Europe, AIME’97 Grenoble, France, March 23–26, 1997 Proceedings, 1997. 261–72.

[20] Bouckaert RR. Bayesian belief networks: from construction to inference. University Utrecht. 1995.

[21] Dai J, Ren J. Unsupervised evolutionary algorithm for dynamic Bayesian network structure learning. In: Advanced Methodologies for Bayesian Networks: Second International Workshop, AMBN 2015, Yokohama, Japan, November 16-18, 2015. Proceedings, 2015. 136–51.

[22] Cooper GF, Herskovits E. A Bayesian Method for Constructing Bayesian Belief Networks from Databases. Uncertainty Proceedings 1991. Elsevier. 1991. 86–94. doi: 10.1016/b978-1-55860-203-8.50015-2

[23] Akaike H. A new look at the statistical model identification. IEEE Trans Automat Contr. 1974;19(6):716–23. doi: 10.1109/tac.1974.1100705

[24] Schwarz G. Estimating the Dimension of a Model. Ann Statist. 1978;6(2). doi: 10.1214/aos/1176344136

[25] Heckerman D, Geiger D, Chickering DM. Learning Bayesian Networks: The Combination of Knowledge and Statistical Data. Machine Learning. 1995;20(3):197–243. doi: 10.1023/a:1022623210503

[26] Geiger D, Heckerman D. Learning Gaussian Networks. Uncertainty Proceedings 1994. Elsevier. 1994. 235–43. doi: 10.1016/b978-1-55860-332-5.50035-3

[27] Kuipers J, Moffa G, Heckerman D. Addendum on the scoring of Gaussian directed acyclic graphical models. 2014.

[28] Seghouane A-K. A note on overfitting properties of KIC and. Signal Processing. 2006;86(10):3055–60. doi: 10.1016/j.sigpro.2006.01.002

[29] Bozdogan H. Mixture-Model Cluster Analysis Using Model Selection Criteria and a New Informational Measure of Complexity. Proceedings of the First US/Japan Conference on the Frontiers of Statistical Modeling: An Informational Approach. Springer Netherlands. 1994. 69–113. doi: 10.1007/978-94-011-0800-3_3

[30] Bozdogan H. Model Selection and Akaike’s Information Criterion (AIC): The General Theory and its Analytical Extensions. Psychometrika. 1987;52(3):345–70. doi: 10.1007/bf02294361

[31] Sclove SL. Application of Model-Selection Criteria to Some Problems in Multivariate Analysis. Psychometrika. 1987;52(3):333–43. doi: 10.1007/bf02294360

[32] Dziak JJ, Coffman DL, Lanza ST, Li R, Jermiin LS. Sensitivity and specificity of information criteria. Brief Bioinform. 2020;21(2):553–65. doi: 10.1093/bib/bbz016 30895308 PMC7299313

[33] Haughton DMA. On the Choice of a Model to Fit Data from an Exponential Family. Ann Statist. 1988;16(1). doi: 10.1214/aos/1176350709

[34] Xu C. Model selection with information criteria. The University of Western Ontario. 2010.

[35] Dean T, Kanazawa K. A model for reasoning about persistence and causation. Computational Intelligence. 1989;5(2):142–50. doi: 10.1111/j.1467-8640.1989.tb00324.x

[36] Murphy KP. Dynamic Bayesian Networks: Representation, Inference and Learning. UC Berkley. 2002.

[37] Margaritis D. Learning Bayesian network model structure from data. Carnegie Mellon University. 2003.

[38] Tsamardinos I, Aliferis CF, Statnikov AR, Statnikov E. Algorithms for large scale Markov blanket discovery. In: FLAIRS conference, 2003. 376–80.

[39] Yaramakala S, Margaritis D. Speculative Markov blanket discovery for optimal feature selection. In: Fifth IEEE International Conference on Data Mining (ICDM’05), 2005. 4-pp.

[40] Tsamardinos I, Aliferis CF, Statnikov A. Time and sample efficient discovery of Markov blankets and direct causal relations. In: Proceedings of the ninth ACM SIGKDD international conference on Knowledge discovery and data mining, 2003. 673–8. doi: 10.1145/956750.956838

[41] Tsamardinos I, Brown LE, Aliferis CF. The max-min hill-climbing Bayesian network structure learning algorithm. Mach Learn. 2006;65(1):31–78. doi: 10.1007/s10994-006-6889-7

[42] Peña JM, Nilsson R, Björkegren J, Tegnér J. Towards scalable and data efficient learning of Markov boundaries. International Journal of Approximate Reasoning. 2007;45(2):211–32. doi: 10.1016/j.ijar.2006.06.008

[43] Li H, Guo H. A Hybrid Structure Learning Algorithm for Bayesian Network Using Experts’ Knowledge. Entropy (Basel). 2018;20(8):620. doi: 10.3390/e20080620 33265709 PMC7513154

[44] Scutari M, Graafland CE, Gutiérrez JM. Who learns better Bayesian network structures: Accuracy and speed of structure learning algorithms. International Journal of Approximate Reasoning. 2019;115:235–53. doi: 10.1016/j.ijar.2019.10.003

[45] Yaramakala S. Fast Markov blanket discovery. In: 2004.

[46] Glover F. Future paths for integer programming and links to artificial intelligence. Computers & Operations Research. 1986;13(5):533–49. doi: 10.1016/0305-0548(86)90048-1

[47] Bollen KA, Harden JJ, Ray S, Zavisca J. BIC and Alternative Bayesian Information Criteria in the Selection of Structural Equation Models. Struct Equ Modeling. 2014;21(1):1–19. doi: 10.1080/10705511.2014.856691 31360054 PMC6663110

[48] Iwok IA, Okpe AS. A comparative study between univariate and multivariate linear stationary time series models. American Journal of Mathematics and Statistics. 2016;6(5):203–12.

[49] Scutari M. Learning Bayesian networks with the bnlearn R package. arXiv preprint. 2009. doi: 10.48550/arXiv.0908.3817

[50] Quesada D, Valverde G, Larrañaga P, Bielza C. Long-term forecasting of multivariate time series in industrial furnaces with dynamic Gaussian Bayesian networks. Engineering Applications of Artificial Intelligence. 2021;103:104301. doi: 10.1016/j.engappai.2021.104301

[51] Nicholson W, Matteson D, Bien J. Bigvar: Tools for modeling sparse high-dimensional multivariate time series. 2017. https://arxiv.org/abs/1702.07094

[52] Akbilgic O. Istanbul stock exchange. UCI Machine Learning Repository. 2013. doi: 10.24432/C54P4J

[53] De Vito S, Massera E, Piga M, Martinotto L, Di Francia G. On field calibration of an electronic nose for benzene estimation in an urban pollution monitoring scenario. Sensors and Actuators B: Chemical. 2008;129(2):750–7. doi: 10.1016/j.snb.2007.09.060

[54] Dua D, Graff C. UCI Machine Learning Repository. 2019. http://archive.ics.uci.edu/ml

